# Effects of Electroacupuncture at Different Acupoints on Functional Dyspepsia Rats

**DOI:** 10.1155/2022/6548623

**Published:** 2022-02-01

**Authors:** Yue-Jie Li, Na-Na Yang, Jin Huang, Lu-Lu Lin, Ling-Yu Qi, Si-Ming Ma, Cheng-Xin Hu, Yu Wang, Jing-Wen Yang, Cun-Zhi Liu

**Affiliations:** ^1^International Acupuncture and Moxibustion Innovation Institute, School of Acupuncture-Moxibustion and Tunia, Beijing University of Chinese Medicine, 11 Beisanhuan East Road, Chaoyang District, Beijing 100029, China; ^2^School of Acupuncture-Moxibustion and Tunia, Beijing University of Chinese Medicine, Shandong 250300, China; ^3^School of Acupuncture-Moxibustion and Tunia, Beijing University of Chinese Medicine, 11 Beisanhuan East Road, Chaoyang District, Beijing 100029, China

## Abstract

Although, acupoint specificity is regarded as the core of scientific issues in electroacupuncture (EA), the difference of EA on treating functional dyspepsia (FD) at different acupoints is unclear. Therefore, this study aims to investigate the different therapeutic effects of EA at lower extremity or abdominal acupoints on the mucosal integrity and lower-inflammatory response in FD. The intragastric administration of iodoacetamide (IA) was performed in 48 rats to establish the FD model. These rats were randomly divided into the control group, the model group and the six EA groups receiving stimulation at the lower extremity (ST36, ST37, and ST39) or abdominal acupoints (ST25, CV4, and CV12) separately. The open-field test (OFT) was measured after 8 weeks of IA, and gastric emptying was evaluated after 10 days of the EA treatment. The local inflammation markers of CD45, eosinophil major basic protein (EMBP), and the tight junction proteins ZO1 and Claudin3 were assessed by immunofluorescence in all groups. Western blot analysis showed that the EMBP and Occludin1 levels in the duodenal. EA at lower extremity acupoint ST36 could improve the gastric emptying. EA at lower extremity acupoints reduced the immunoreactivity of EMBP, but the CD45 was reregulated by the ST37 and ST39 acupoints. The lower extremity acupoints also ameliorated FD-tight junction protein in the expression of Claudin3 and ZO1. However, only the ST36 suppressed the expression of EMBP and recovered the expression of Occludin1. Similarly, the effect of EA at abdominal acupoints was not obvious either in facilitating gastric motility or in improving inflammatory and mucosal injury. EA at lower extremity and abdominal acupoints with the same stimulation parameters had different therapeutic effects in gastric emptying, intestinal mucosal integrity, and inflammation response, thus proving the specificity of acupoints.

## 1. Introduction

Functional dyspepsia (FD), the prevalent gastrointestinal disorder, is the presence of symptoms originating from the gastroduodenal region in the absence of any organic, systemic, or metabolic disease [1]. It affected 8% to 12% of the worldwide population and substantially impaired the quality of life. Furthermore, the duration of FD has a major financial impact, and the annual additional healthcare costs related to FD have been estimated at 18$ billion in the USA [2]. Despite the common occurrence of FD with considerable healthcare expenses and impact on the quality of life, the current treatment options are limited. For example, domperidone has an obvious peripheral blocking effect and could inhibit the occurrence of nausea and vomiting, but it was easy to recur due to discontinuation of the medication. Proton pump inhibitors (PPIs) were often accompanied by gastrointestinal adverse reactions such as abdominal pain and diarrhea [3]. Consequently, effective and convenient treatment with a low risk of adverse effects remains to be developed.

Although structural diseases were an exclusion criterion for FD, recent advance in research show that in a proportion of case of FD, there are tangible but subtle disorders of gut function and immunological disorders, which may be amenable to therapies aimed at the disease rather than at symptom relief [4]. Growing evidence indicated that FD is associated with duodenal disease, including the expansion of activated eosinophils (EOS) in the duodenal, which could cause immune pathology and damage the intestinal mucosal integrity. Therefore, eosinophils in the duodenum are increasingly accepted as key players in the pathogenesis of dyspepsia [5, 6].

Acupuncture may be a potentially effective nonpharmacologic therapy for functional gastric disorders [7, 8]. Our previous randomized controlled trial has shown that acupuncture was an effective and safe method to relieve postprandial distress syndrome, with pretty long-term outcomes [2]. Actually, functional dyspepsia is frequently thought of as an abnormal condition of the stomach, which had been widely recognized. Until recently, changes in the duodenal are receiving increasing attention. In addition to CV4 and CV12, each acupoint belongs to the stomach meridian of the twelve meridians, and is widely used in the clinical treatment of various gastrointestinal diseases. Both the CV4 and CV12 are located on the abdomen, which conforms to the principle of proximal acupoint selection. ST36 is “converging point,” ST25 is the “front-mu point” of large intestinal meridian, ST38 and ST39 are the “lower confluent acupoints” of large intestinal meridian and small intestinal meridian, respectively. Rising studies indicated that different acupoints influence the therapeutic effects of acupuncture [2]. Acupoints at the lower extremity and abdominal acupoints are widely applied in functional gastrointestinal disorders, but study argued that electroacupuncture in the abdomen inhibited gastrointestinal motility [10]. Besides, Ma and his colleagues reported that low-intensity EA at abdominal acupoints cannot generate anti-inflammatory effects after LPS model [11]. Therefore, this study aims to investigate the difference of electroacupuncture employed at the lower extremities and abdominal acupoints on the symptom of FD rats.

## 2. Materials and Methods

### 2.1. Animals

Specific-pathogen-free (SPF) male Sprague-Dawley 10-day-old rats were purchased from the Charles River Laboratory Animal Technology Co. Ltd. (Beijing, China). The rats were housed under the following conditions: the temperature of the feeding environment was 22 ± 2°C, with a relative humidity of 50%, and 12 light/dark cycle. All procedures had followed the guidelines of the Statute on the Administration of Laboratory Animals. The Institutional Animal Care and Use Committee (IACUC) approved animal activities under the approval number BUCM-2019101801-4019.

### 2.2. FD Model and Animal Grouping

An established model of FD by LIU [9] was used in this experiment. Briefly, rats were given a gavage of 0.1% iodoacetamide in 2% sucrose daily for six days, and then kept in cages with free water and food until they were eight weeks old. After eight weeks, 60 rats were randomly divided into 8 groups: the control group (*n* = 12), the model group (*n* = 12), and the 6 groups (*n* = 6) receiving EA treatment at bilateral ST25 (Tianshu), ST36 (Zusanli), ST37 (Shangjuxu), ST39 (Xiajuxu), and unilateral CV4 (Guanyuan), CV12 (Zhongwan), respectively.

### 2.3. EA Intervention

**Figure 1:**
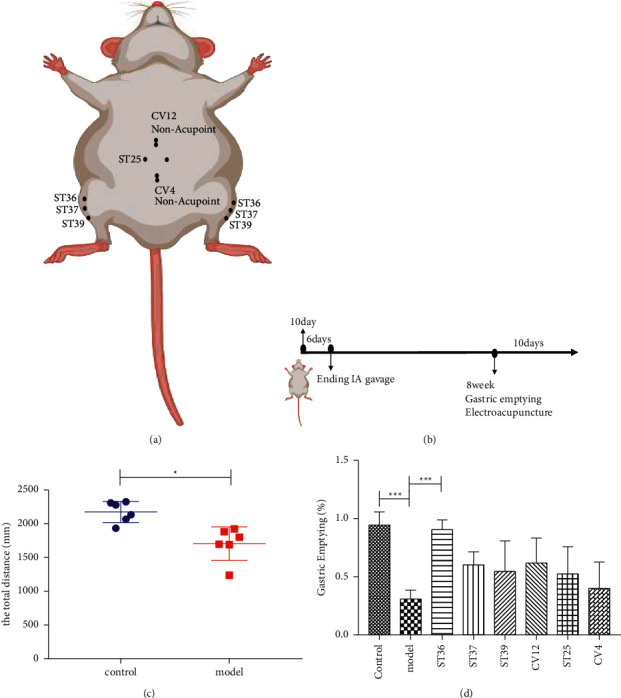
Gastric emptying (%) was differences at EA stimulation abdominal and lower extremity acupoints. The acupoints diagram and important time points ((a, b); the distance of animal explored (c); gastric motility of FD model rats and the EA treatment group was evaluated by gastric emptying (d). all ^*∗*^*p* < 0.05, ^*∗∗*^*p* < 0.01, compared with the FD model group, *n* = 6.

All rats were immobilized in a black controller, with the central part of the abdomen and the four limbs expose without anesthesia, EA intervention was applied for once a day, 30 min lasting for 10 days. After routinely disinfecting the sterile stainless steel acupuncture needles (0.22 mm × 25 mm, Beijing ZhongYan Taihe Medical instrument Co. Ltd., Beijing, China), they were vertical inserted into the skin lasting for 30 min every day. One pair of sterile acupuncture needles was inserted bilaterally to acupoints ST36, ST37, ST38, and ST25, respectively. As for the unilateral acupoints CV4, the needle penetrated into the skin of CV4, a second needle was inserted perpendicularly to a depth of 1 mm at a nonacupoints 1 mm lateral to CV4 to from a pair for EA. The CV4 performed the same operation as CV12 (Table 1 and Figure 1(a)). The needles were connected to an EA apparatus (HANS-200A Nanjing, China) and stimulated at the frequency of 100 Hz via a continuous wave and the intensity of 1 mA. The location of each acupoints is listed in Table 1. The control group and the model group remain immobilized for 30 min.

### 2.4. Open-Field Test (OFT)

The OFT was performed according to Lin. In a dark and quiet room, the rats belonging to the control group and the model group were placed at the center of box (100 cm *∗* 100 cm *∗* 50 cm) one by one and allowed to explore for 3 min, and then the animal dung was wiped and the box dealt with 75% alcohol. We collected the data for analysis, i.e., the total distance (Figure 1(b)).

### 2.5. Gastric Emptying

In FD rats, gastric emptying of a solid meal was measured as described with modification [10]. In a word, overnight fasting rats were given preweighed 30 g food normal feeding for 3 h with free access to water. Under anesthesia with high concentration isoflurane, animals quickly lost their breathing and heartbeat after 3 h. Open the abdominal cavity and remove fresh duodenal tissue, then the stomachs were removed quickly and weighed thoroughly. The amount of food contained in the stomach was different between the total weight of the stomach before and after stomach emptying. The speed of gastric emptying for the entire 3 h period was calculated as follows: gastric emptying (%) = 100 − (gastric content/food intake) × 100%.

### 2.6. Immunofluorescence

Under anesthesia with high concentration isoflurane, animals quickly lost their breathing and heartbeat. Open the abdominal cavity and remove fresh duodenal tissue. Part of tissues were rinsed with saline and immersed in 4% paraformaldehyde for 24 hours. Gradient dehydrated in 20% and 30% sucrose in 0.01 PBS at 4°C for 24 hours. Sections of 6 *μ*m were deparaffinized following general procedures, blocking with the bovine serum for 1 h at 37°C, and incubated with rat antimouse primary antibodies ZO-1 (1 : 2000; Abcam), claudin3 (1 : 2000; Proteintech), CD45 (1 : 2000; Proteintech), and EMBP (1 : 2000; Santa Cruz Biotechnology) at 4°C overnight. Subsequently, sections were incubated for 1 h at room temperature in the dark with donkey antirabbit secondary antibody (1 : 2000; Abcam). Cell nuclei were stained with 4′,6-diamidino-2-phenylindole (DAPI; ORIGENE), and the cover glass was enclosed onto the paraffin sections. Photomicrographs were obtained using a microscope (Olympus, Tokyo, Japan). We chose at least 6 representative nonoverlapping high-power field at ×400 magnification in a blinded manner. The number of claudin3 and the number of ZO1, CD45, EMBP-positive cells were quantified using the ImageJ program (National Institutes of Health).

### 2.7. Western Blot

Duodenal tissues were ground and lysed in RIPA buffer, and 40 *μ*g total protein was separated by 12% SDS-PAGE, and then transferred to the polyvinylidene difluoride membrane. The blots were blocked with 5% skimmed milk powder in PBST and incubated overnight at 4°C with the anti-Occludin1 (1 : 1000, Proteintech Group), anti-EMBP (1 : 500, Santa Cruz Biotechnologies), and anti-*β*-actin (1 : 5000, Proteintech Group). The membrane was incubated with secondary antibodies in room temperature 1 h. The proteins were imaged by the ChemiDoc imaging system (Biorad, Hercules, CA, USA) after the addition of a chemiluminescent substrate.

### 2.8. Statistical Analysis

All data were presented as mean ± S.E.M. Paired and unpaired student's *t*-test and one-way and two-way analysis of variance (ANOVA) with post hoc tests for Tukey's multiple comparisons were used to assess the effects of EA on different acupoints. *P* < 0.05 was considered to be statistically significant. Statistical analyses were performed using GraphPad Prism 8.0 (GraphPad Software, San Diego, CA, USA).

## 3. Results

### 3.1. The Effects of EA at the Different Acupoints on the Gastric Motility

We assessed the model of FD by open field test (OFT). The distance of locomotion in the model group was significantly lower (*P* < 0.05) than that in the control group, which proved that the model group showed the emotional symptoms (Figure 1(c)).

The gastric motility of rats was measured by gastric emptying, and the motility of gastrointestinal delayed on the model rats. Compared with model group, the gastrointestinal motility of rats was affected through EA treatments. EA at the lower extremities (ST36) significantly increased the motility of gastric emptying (Figure 1(d)) (*P* < 0.05). On the contrary, the effects of EA at abdominal acupoints (ST25, CV4, or CV12) on gastric emptying were not obvious.

### 3.2. The Effect of EA at the Lower Extremity and Abdominal Acupoints Were Different on the Inflammatory of the Duodenal Mucosa

**Figure 2:**
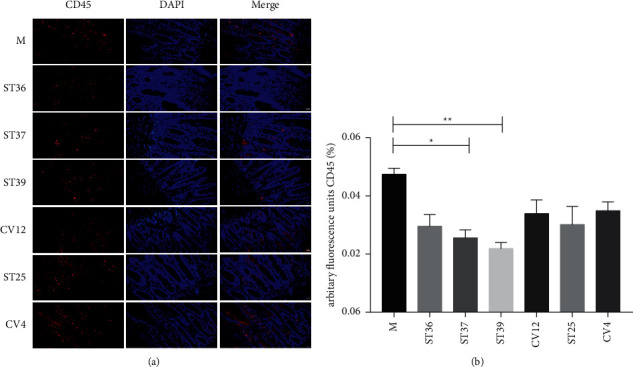
The difference of EA at lower extremity and abdominal acupoints on the inflammatory response of intestinal mucosal. (a) Typical results of immunofluorescence staining to detect the expression of CD45 (red). Blue signals indicate DAPI nuclear staining (scale bar, 20 *μ*m); (b) the percentage of CD45 in duodenal mucosa (% of marked number). The results of quantification are expressed as the mean ± SEM of at 6 photos. ^*∗*^*p* < 0.05, ^*∗∗*^*p* < 0.01 compared with the FD model group, *n* = 6.

Low-grade inflammation of duodenal mucosa plays a key role in the pathogenesis of FD. To determine whether electroacupuncture can improve the infiltration of immune cells in the duodenal of FD rats, we used CD45 to detect the number of leukocytes in the duodenal mucosa of rats in each group. A significant increase in the amount of CD45 was observed in the duodenal (Figure 2(a)). EA at ST37 or ST39 significantly reduced the activity of CD45 expression compared with the model group (Figures 2(a) and 2(b)). However, EA at ST36, ST25, CV4, or CV12 acupoints had a mild decrease in CD45 expression, it was no significant (Figure 2(b)) (*P* > 0.05).

**Figure 3:**
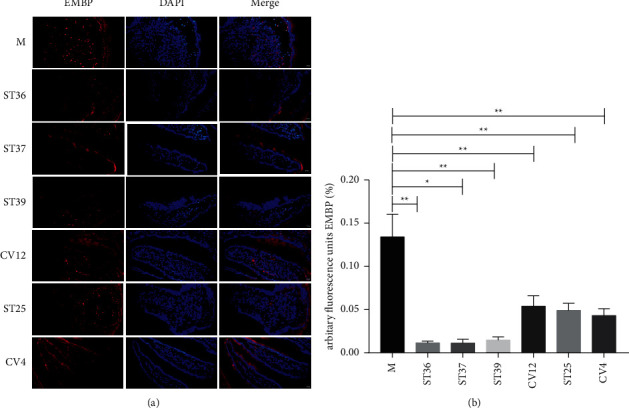
The difference of EA at lower extremity and abdominal acupoints on the content of EMBP. (a) Typical results of immunofluorescence staining to detect the expression of EMBP (red). Blue signals indicate DAPI nuclear staining (scale bar, 20 *μ*m); (b) the percentage of EMBP in duodenal mucosa (% of marked number). The results of quantification are expressed as the mean ± SEM of at 6 photos. ^*∗*^*p* < 0.05, ^*∗∗*^*p* < 0.01 compared with the FD model group, *n* = 6.

Based on the finding of leukocyte infiltration in the duodenum of FD rats, we further explored the effect of EA on the activation of eosinophil (Figure 3). In the duodenal (Figure 3(a)), immunostaining of EMBP was obviously stronger in the model group. However, when intervened with EA, the fluorescence intensity was weakened compared with model rats (Figure 3(b)) (*P* < 0.05). Importantly, we also found that EA at lower extremity could reduce obviously the level of EMBP.

**Figure 4:**
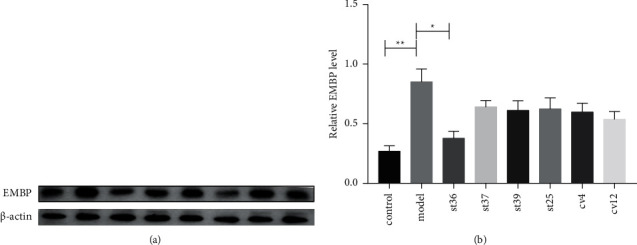
The effect of EA on the expression of EMBP on FD rats. (a) The expression of EMBP; (b) the expression of EMBP. The results of quantification are expressed as the mean ± SEM. ^*∗*^*p* < 0.05, ^*∗∗*^*p* < 0.01 compared with the FD model group, *n* = 6.

To further prove our observation, we used the Western blot analysis that also revealed remarkable increased expression of the EMBP protein in the FD model group compared with that observated in the control group (Figure 4(a)) (*P* < 0.05). EA treatment at ST36 significantly reduced the level of EMBP compared to other acupoint groups, which showed ST36 was the appropriate acupoint for improving the inflammation of FD (Figure 4(b)).

### 3.3. The Difference of EA at the Lower Extremity and Abdominal Acupoints on the Duodenal Mucosal Integrity

**Figure 5:**
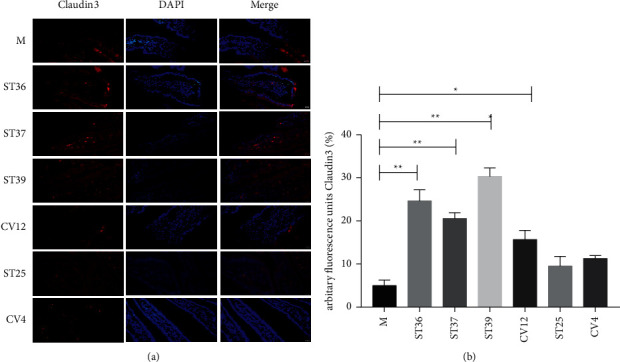
The difference of EA at lower extremity and abdominal acupoints on the content of Claudin3. (a) Typical results of immunofluorescence staining to detect the expression of Claudin3 (red). Blue signals indicate DAPI nuclear staining (scale bar, 20 *μ*m); (b) the percentage of Claudin3 in duodenal mucosa (% of marked area). The results of quantification are expressed as the mean ± SEM of at 6 photos. ^*∗*^*p* < 0.05, ^*∗∗*^*p* < 0.01 compared with the FD model group, *n* = 6.

Persistent low-grade duodenal inflammation can destroy the integrity of its mucosa and damage its function. Therefore, we continued to explore the effect of EA at different acupoints on mucosal integrity. Recent findings have demonstrated that Claudin, Oclaudin, and ZO1 were implicated in the regulation of the mucosa barrier, which were abnormal in FD patients and animal models. We performed immunofluorescence to detect the expression levels of the tight junction proteins Claudin3 and ZO1. As shown in Figure 5(a), the number of Claudin3 was upregulated, and the expression of Claudin3 was apparently stronger in ST36, ST37, or ST39 compared with the model group (*P* < 0.05). Nevertheless, the fluorescence intensity was mildly enhanced after the intervention of CV4, CV12, or ST25, which had no statistical significance (Figure 5(b)) (*P* > 0.05).

**Figure 6:**
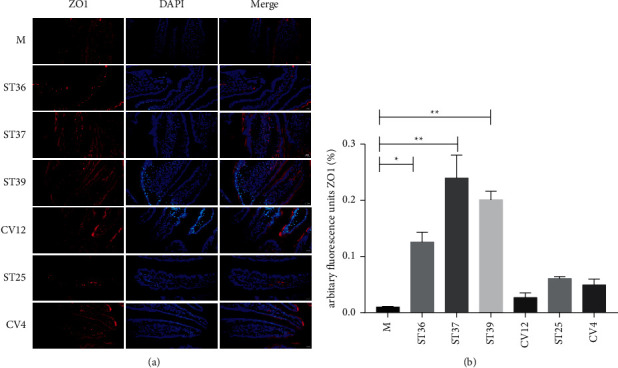
The effect of EA on the expression of ZO1 on FD rats. (a) Typical results of immunofluorescence staining to detect the expression of ZO1 (red). Blue signals indicate DAPI nuclear staining (scale bar, 20 *μ*m); (b) the percentage of ZO1 in duodenal mucosa (% of marked number). The results of quantification are expressed as the mean ± SEM of at 6 photos. ^*∗*^*p* < 0.05, ^*∗∗*^*p* < 0.01 compared with the FD model group, *n* = 6.

Moreover, we observed that there was dropped off ZO1 expression in the model group (Figure 6(a)). On the contrary, the ZO1 positive staining was obviously increased after EA treatment, particularly in the lower extremity acupoints (ST36, ST37, or ST39) groups compared with the model group (Figure 6(b)). The abdominal acupoints seem to have no effect on the expression of ZO1. These findings were consistent with the mucosa reparability determined by immunofluorescence analysis of Claudin3.

**Figure 7:**
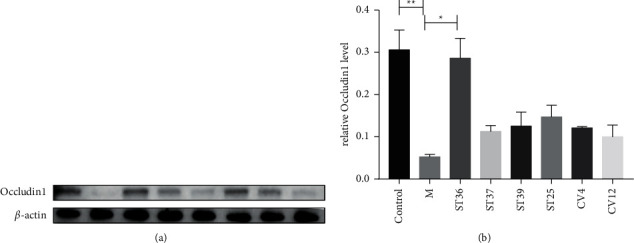
The effect of EA on the expression of Occludin1 on FD rats. (a) The expression of Occludin1; (b) the expression of Occludin1. The results of quantification are expressed as the mean ± SEM. ^*∗*^*p* < 0.05, ^*∗∗*^*p* < 0.01 compared with the FD model group, *n* = 6.

To further investigate which acupoints are effective for promoting the recovery of FD mucosal barrier function, western blot analysis was carried out to show the effects of EA at different acupoints in rats. A remarkable difference is found in the expression of Occludin1 between the control group and the FD model group (Figure 7(a)). An improvement was observed in the ST36 group, whereas there was no significant increase in the other acupoints groups (Figure 7(b)) (*P* < 0.05).

This finding was consistent with the trend of tight junction protein determined by immunofluorescence analysis of Claudin1 and ZO-1 staining.

## 4. Discussion

This study suggested that EA at different acupoints had specific effect on gastric motility, duodenal low-grade inflammation, and duodenal mucosal integrity in functional dyspepsia. Only the lower extremity acupoint ST36 promoted the gastric motility, played mild anti-inflammation, as well as upregulated the expression of tight junction protein in the duodenal mucosal.

The gastrointestinal insufficiency was one of the main symptoms of FD patients. The reduced of gastric motility was proved the FD model was succussed. At the same time, communication between the central nervous and the enteric nervous system had been appreciated, and the fact that brain-gut communications were bidirectional had been realized recently. In this study, we used a previously established rodent model of FD which only were gavage with iodoacetamide whereases not others manipulation. Our results indeed showed that the model reduced the distance of movement, suggesting that there were developed emotional symptoms. The symptoms were highly consistent with the clinical emotional of FD patients, which was laterally verified to be successful of the model. A decrease in gastric motility and tight junction protein expression indicated that duodenal acidification damage the intestinal barrier [14]. At the same time, intestinal barrier dysfunction was believed to be a key risk factor for intestinal inflammation, and increasing intestinal permeability was a potential pathogenic mechanism that could be involved in the generation of low-grade duodenal inflammation and symptoms in FD [6, 15]. Therefore, we evaluated the presence of low-grade inflammation and measured the expression of adhesion proteins and duodenal mucosal barrier function on FD models after different acupoints EA treatment.

Duodenal inflammation was closely related to the pathogenesis of FD, and the expression of inflammatory cytokines was reduced by EA [16]. The destruction of the duodenal mucosal barrier and the low grade of mucosal inflammation mainly caused by local EOS immune activation might play an important role [17]. The release of EOS after degranulation produced a variety of substances, including EMBP, which acts on the intestinal epithelium and destroys its integrity. Our study indicated that EA could significantly reduce the expression of EMBP in intestinal tissue no matter electroacupuncture at the lower extremity or abdominal acupoints with the high-intensity (2 mA). There were, however, a number of reasons why it is difficult to treat leukocytes, one of which might be considered as CD45 lacked specificity.

As a traditional Chinese therapy, EA had been used to regulate the content of EOS. Carneiro ER [16] showed that acupuncture can reduce the number of EOS in BALF of asthma model, and the regulation of EOS by acupuncture is positively correlated with interleukin-4 (1L-4) and interleukin-10 (1L-10), but negatively correlated with the production of interleukin-1 (1L-1) and interferon-*γ* (IFN-*γ*), suggesting that acupuncture may regulate EOS to balance intercellular TH1/TH2 immune response. The regulation of EOS function is probably one of the main mechanisms of acupuncture in the treatment of FD. However, the regulatory effect and mechanism of acupuncture on the differentiation, development, migration, and accumulation of EOS in the inflammatory region of FD need to be further studied. The damage of the intestinal mucosal barrier especially the mechanical barrier may be an important pathogenesis of relapse and delay of FD. The study showed that there was mild inflammation in the duodenum of patients with FD that led to a decrease in the expression of intestinal epithelial tight junction proteins claudin3, ZO1, resulting in abnormal structure and function of intestinal epithelial tight junction proteins, and causing the dysfunction of intestinal mucosal barrier eventually [11, 12].

Studies showed that EA could adjust the structure of tight junction protein and had an obvious therapeutic effect. The experimental study showed that the expression of claudin and ZO1 of tight junction protein in the obese rat model increased. At the same time, it was found that it could effectively improve the intestinal mucosal barrier function by inhibiting colonic inflammation, and then reducing the expression of claudin and ZO1 [13]. The results showed that after treatment, the inflammatory factors IL-6 and TNF-*α* decreased in FD rats, which could play a therapeutic role in FD by improving the integrity of tight junction protein, reducing intestinal mucosal permeability, and improving low-grade duodenal inflammation [14].

Acupoint specificity regulation was considered as an important characteristic of acupuncture in Chinese medicine. It had been defined as the difference of attending function of different acupoints in the same meridian.

This study indicated that FD impaired duodenal tight junction protein and increased mucosal barrier permeability. EA treatment could repair the damaged mucosal barrier to varying degrees, and the effect of lower extremity acupoints is better. After EA treatment, the expression of tight junction protein increased in different degrees in FD rats, and the lower extremity (ST36 group, ST37 group, ST39 group) upregulated the content of Claudin3 and ZO1significantly. The ST36 increased the expression of Occludin1 that manifested different acupoints played different roles and functions in the treatment of FD. Abdominal acupoints and lower extremities acupoints have different therapeutic effects, strongly demonstrating the specificity of acupoints.

In addition, improving gastric emptying was similar to those in repairing duodenal mucosa in this study. In a previous study, we accessed that EA with different acupoints accelerated gastric motility that had different effects in the postoperative ileus mice [15]. The EA at ST36 improved the rhythm in the rats, patients' stomachs, and regulated gastric emptying [16]. Consistently, this study also proved that EA at ST36 had the function of regulating gastric motility. However, the abdominal acupoints especially the ST25 as a common acupoint for the clinical treatment of gastrointestinal diseases, and we firmly believe its therapeutic effect. The ST25 had the potential therapeutic for gastric motility, which could improve the symptom of FD traditionally. In our study, we only observed the ST25 with 100 Hz, 1 mA had no significantly therapeutic. We could not be sure that changing the parameters would have produced different results. A number of studies proved the parameters of electroacupuncture are crucial, which decided the effect of treatment sometimes. Sato and his colleagues thought that gastric motility was often excited when the acupoints on the limbs were stimulated. Our preliminary research, we found that ST25 with different parameters had different effect. The ST25 with 2/100 Hz could improve the motility of postoperative ileus whereas not 2 Hz, 15 Hz, and 100 Hz.

Interestingly, the specificity of acupoints might have different manifestations in different conditions. The different responses of these regions to different acupoint stimulation might be related to the acupoint's specificity. Recent studies demonstrated that EA at ST37 increased IL-4 and IL-1*β* in ulcerative colitis model rats, which is beneficial to eliminate inflammatory reactions and repair damaged tissue and has a certain therapeutic effect on ulcerative colitis [17, 18]. Studies have reported that EA at ST39 increased the level of TNF-*α* in intestinal tissue and the injury of duodenal mucosa [19, 20]. Moreover, the lower extremity acupoints were more effective in repairing duodenal mucosa and improving gastric motility than abdominal acupoints in the FD models. EA at different acupoints had different effects on improving gastric motility, impairing intestinal permeability, and controlling the progression of inflammation. As we all know, ST36, ST37, and ST39 are all located in the stomach meridian, and from the close physiological function between the stomach, large and small intestinal, there is more mutual influence between batches in the pathological condition. Acupoint locations had different sensitives to EA due to the different stimulation intensities. The high intensity at the abdominal acupoints maybe drive a spinal-sympathetic axis, promoting anti-inflammatory effects that depend on NPYDBH-marked noradrenergic neurons. However, the high intensity at the lower extremities acupoints could also drive a spinal-sympathetic axis and produce vagal efferent independent anti-inflammatory effects. Some studies showed that vagal and sympathetic pathways associated with gastric motility regulate can be evoked from lower extremities and abdominal severally [10, 11]. Therefore, these findings indicated that the surface lower extremity and abdominal acupoints are relatively specific in the treatment of FD. EA at the ST36 could improve the gastric motility, restore mucosal integrity, and reduce the inflammation of FD rats.

## 5. Conclusion

In conclusion, we confirmed that EA at lower extremities acupoints (ST36, ST37, ST39) and abdominal acupoints (ST25, CV4, CV12) played different roles in the treatment of FD. Mechanistically, EA at ST36 potentially suppressed the low-grade inflammation in the duodenal to increase the integration of duodenal mucosal, in turn, to improve FD symptoms.

## Figures and Tables

**Table 1:** The location of acupoints in the study.

Acupoints	Location	Operation
ST36 (Zusanli)	Lower lateral knee joint, 3.5 mm below fibula head	Needle perpendicularly with the depth 4-5 mm
ST37 (Shangjuxu)	ST37 was located about 6 mm below ST36 on the hind leg	Needle perpendicularly with the depth 4-5 mm
ST39 (Xiajuxu)	ST39 was located about 12 mm below ST36 on the hind leg	Needle perpendicularly with the depth 4-5 mm
ST25 (Tianshu)	Located lateral to the umbilicus, at the mid-point from the umbilicus to the nipple line	Needle perpendicularly with the depth 5 mm
CV4 (Guanyuan)	Located at the point of 3/5 down the ventral midline connecting the umbilicus to the pubic tubercle	Needle perpendicularly with the depth 5 mm
CV12 (Zhongwan)	Located at the mid-point of the ventral midline connecting the umbilicus to the sternum	Needle perpendicularly with the depth 5 mm

## Data Availability

The data used to support the findings of this study are included within the article.

## References

[1] Ford A. C., Mahadeva S., Carbone M. F., Lacy B. E., Talley N. J. (2020). Functional dyspepsia. *The Lancet*.

[2] Yang J.-W., Wang L.-Q., Zou X. (2020). Effect of acupuncture for postprandial distress syndrome. *Annals of Internal Medicine*.

[3] Masuy I., Van Oudenhove L., Tack J. (2019). Review article: treatment options for functional dyspepsia. *Alimentary Pharmacology & Therapeutics*.

[4] Enck P., Azpiroz F., Boeckxstaens G. (2017). Functional dyspepsia. *Nature Reviews Disease Primers*.

[5] Talley N. J., Walker M. M., Aro P. (2007). Non-ulcer dyspepsia and duodenal eosinophilia: an adult endoscopic population-based case-control study. *Clinical Gastroenterology and Hepatology*.

[6] Vanheel H., Vicario M., Vanuytsel T. (2014). Impaired duodenal mucosal integrity and low-grade inflammation in functional dyspepsia. *Gut*.

[7] Lim H.-D., Kim M.-H., Lee C.-Y., Namgung U. (2016). Anti-inflammatory effects of acupuncture stimulation via the vagus nerve. *PLoS One*.

[8] Balestrini J. L., Tsuchida D., Fukuda H., Pappas T. N., Takahashi T. (2005). Acupuncture accelerates delayed gastrointestinal transit after abdominal surgery in conscious rats. *Scandinavian Journal of Gastroenterology*.

[9] Liu L. S., Winston J. H., Shenoy M. M., Song G. Q., Chen J. D. Z., Pasricha P. J. (2008). A rat model of chronic gastric sensorimotor dysfunction resulting from transient neonatal gastric irritation. *Gastroenterology*.

[10] Dai F., Lei Y., Li S., Song G., Chen J. D. Z. (2013). Desvenlafaxine succinate ameliorates visceral hypersensitivity but delays solid gastric emptying in rats. *American Journal of Physiology-Gastrointestinal and Liver Physiology*.

[11] Taki M., Oshima T., Li M. (2019). Duodenal low-grade inflammation and expression of tight junction proteins in functional dyspepsia. *Neuro-Gastroenterology and Motility*.

[12] Chen J., Xuan Y.-h., Luo M.-x. (2020). Kaempferol alleviates acute alcoholic liver injury in mice by regulating intestinal tight junction proteins and butyrate receptors and transporters. *Toxicology*.

[13] Wu H., Liang F. X., Chen B. G., Chen L. (2019). [Effects of electroacupuncture on inflammatory response and intestinal mucosal barrier in obese rats with insulin resistance]. *Zhongguo Zhen Jiu*.

[14] Chang X., Zhao L., Wang J., Lu X., Zhang S. (2017). Sini-san improves duodenal tight junction integrity in a rat model of functional dyspepsia. *BMC Complementary and Alternative Medicine*.

[15] Yang N. N., Ye Y., Tian Z. X. (2020). Effects of electroacupuncture on the intestinal motility and local inflammation are modulated by acupoint selection and stimulation frequency in postoperative ileus mice. *Neuro-Gastroenterology and Motility*.

[16] Chang C.-S., Ko C.-W., Wu C.-Y., Chen G.-H. (2001). Effect of electrical stimulation on acupuncture points in diabetic patients with gastric dysrhythmia: a pilot study. *Digestion*.

[17] Shi Y., Li T., Zhou J. (2019). Herbs-partitioned moxibustion combined with acupuncture inhibits TGF-*β*1-smad-snail-induced intestinal epithelial mesenchymal transition in Crohn’s disease model rats. *Evidence-Based Complementary and Alternative Medicine*.

[18] Yu Q. H., Li L. H., Lu Y. W. (2020). [Moxibustion combined with acupoint catgut embedding promotes recovery of injured colonic mucosa by suppressing inflammation in ulcerative colitis rats]. *Zhen Ci Yan Jiu*.

[19] Ling X., Zhang H., Yi X. Q., Wu J. F. (2016). [Effects of electroaupuncture stimulation of “Xiajuxu” (ST 39), etc. on duodenal mucosal injury, serum pro-inflammatory factors levels and duodenal nicotinic acetylcholine receptor alpha 7 expression in duodenal ulcer rats]. *Zhen Ci Yan Jiu*.

[20] Zhang H., Wang Z. Z., Zhang Y. C. (2015). [Effect of electroacupuncture of “Xiaohai” (SI 8) and “Xiajuxu” (ST 39) on serum TNF-*α* and duodenal high mobility group protein B 1 levels in duodenal ulcer rats]. *Zhen Ci Yan Jiu*.

